# Comparative Effect of Multi-Dose Contrast Median on Contrast-Enhanced Computed Tomography Workflow of Nurses and Hospital Efficiency: A Multi-CenterReal-World Prospective Observational Study in China

**DOI:** 10.1155/2022/1168973

**Published:** 2022-12-13

**Authors:** Chen Zhang, Zhilei Fan, Feng Ma, Da Li, Yingyao Chen, Yan Wei

**Affiliations:** Key Laboratory of Health Technology Assessment, National Health Commission, School of Public Health, Fudan University, Shanghai, China

## Abstract

**Objective:**

This study aims to evaluate and compare computed tomography (CT)-contrast operational workflow and hospital imaging efficiency when using a multi-dose bulk IV contrast delivery system and when using a single-dose packaging contrast.

**Materials and Methods:**

A multi-center prospective observational study was conducted in six regions in China. The operating time and workflow of radiology nursing staff were evaluated and observed using an investigational tool and recorded by the investigators using a stopwatch. Nursing staff's knowledge and the imaging capabilities of hospitals were collected using a questionnaire. Rate, *t*-test, *χ*^2^ test, and partial correlation analysis were used to describe the knowledge of nursing staff. The operation time and frequency of the two contrast agent packages were further compared using the Stata 15.0 software.

**Results:**

A total of 42 radiology nurses and 1,167 CT contrast-operating procedures in six provinces in China were evaluated. The total operating times for the 100 ml contrast agent versus the 200 ml contrast agent were 80.67 s and 63.81 s, respectively (*P* < 0.01). According to the average annual hospital CT scans (49,807 scans) and the power injector (PI) market share, approximately 233 h yearly could be saved in a hospital. Regarding CT contrast knowledge, approximately 57.14% nurses expressed their willingness to use multi-dose packaging contrast agents.

**Conclusion:**

Through difference and correlation analysis on real-world data, this study suggests that, considering safety, the use of a multi-dose bulk IV contrast agent is more time-saving and efficient for Chinese nurses and medical institutions compared with that of a single-dose package.

## 1. Introduction

Several health technologies including computed tomography (CT), magnetic resonance imaging (MRI), positron emission tomography and CT (PET/CT), and PET/MRI can be used for medical diagnostic imaging. Among these technologies, CT plays an essential role in diagnostic imaging, especially in the assessment of bones and calcifications [1]. Despite the increasing rate of CT supply in China, there are still huge gaps between the number of CT and MRI scanners per million population in China compared with the Organisation for Economic Co-operation and Development (OECD) countries [2]. Meanwhile, the demand for CT diagnosis is high. Moreover, the number of annual CT examinations has increased gradually and yearly over the last decades [3]. Unlike unenhanced CT, intravenous (IV) contrast-enhanced CT (CECT) features a contrast agent, a pharmaceutical agent that can improve diagnostic image quality, consequently increasing accuracy for disease diagnosis.

Nurses in Chinese hospitals constantly work in a high-intensity work environment, typically with long working hours weekly during both day and night shifts [4]. High-intensity workloads and burnout affect nurses on a daily basis, jeopardizing their job performance, sleep quality, and—indirectly—patient safety [5, 6]. Nursing in the radiology department is particularly complex and stressful: radiology nursing duties often include mandatory overtime, which are attributable to the cumbersome operations involved in handling contrast agents [7]. Generally, contrast agents are diverse in contrast dose standards and packages. Currently, single-dose packaging of the 50 and 100 ml vials makes up the majority of contrasts used in IV contrast-enhanced CT in China, while multi-dose IV contrast media (in 200 and 500 ml bulk packages) remain more seldomly used. Multi-dose packaging contrast, however, has many known benefits. It not only simplifies the operations process for the nursing staff but also reduces the medical cost for patients through medical reimbursement and improves the CT scanning efficiency in hospitals. Previous studies on IV contrast-enhanced computed tomography (CECT) mainly examine three relevant topics: (1) the microbiologic contamination and time efficiency of using different power injectors (PIs) [8–11]; (2) contrast cost savings and waste evaluation using a multi-dose bulk IV contrast system [12, 13]; and (3) cost savings of using multi-dose packaging contrast media together with reimbursement units for patients undergoing IV contrast-enhanced CT [14].

This study aims to evaluate CT-contrast operational workflow and hospital imaging efficiency when using a multi-dose bulk IV contrast delivery system (under two types of PIs) compared with a single-dose packaging contrast. Specifically, this analysis is aimed at evaluating the nursing staff's knowledge, operating time saved, and scanning efficiency improvements resulting from the replacement of 100 ml single-use IV contrast vials (350 mg/ml iodine concentration) with a multi-dose bulk IV contrast delivery system (350 mg/ml iodine concentration). This study was conducted from the Chinese nurses' and medical institutions' perspectives, accordingly providing evidence, countermeasures, and references for decision making in hospitals in China.

## 2. Materials and Methods

### 2.1. Real-World Data Collection

#### 2.1.1. Observational Study Design

A multi-center prospective observational study was conducted between October and November 2018 in six regions in China, including East China (Zhejiang Province and Anhui Province), North China (Hebei Province), Central China (Hunan Province and Hubei Province), South China (Guangdong Province), Northeast China (Liaoning Province), and Northwest China (Shaanxi Province). One tertiary hospital was selected per province, and a total of eight tertiary hospitals were surveyed for real-world data collection. Nurses were assigned to different CT scanner rooms in each observation and followed up for one to three days (mostly from 7:30 AM to 7:30 PM). The investigators further recorded the operating time and workflow of the nurses. The investigators also collected information on the nurses' basic characteristics as well as their understanding, attitude, willingness to use, and knowledge about the multi-dose packaging contrast.

In the study, nursing staff used either 100 ml single-use IV contrast vials (350 mg/ml iodine concentration) or a multi-dose bulk IV contrast delivery system (350 mg/ml iodine concentration, 200 ml). Two PIs, i.e., syringeless and dual-syringe PIs, were considered in this study. Accordingly, four strategies were generated: (1) 100 ml and syringeless PI, (2) 100 ml and dual-syringe PI, (3) 200 ml and syringeless PI, and (4) 200 ml and dual-syringe PI. Moreover, based on the analysis of the 200 ml sample, this study simulated the scenario of using a 500 ml product contrast agent.

#### 2.1.2. Evaluation of Nursing Staff's Knowledge

A 5-point Likert scale was used to evaluate the nursing staff's degree of understanding, attitude, and intention of use toward the multi-dose packaging contrast (i.e., 1 for “very poorly understood/unsupportive/unwilling,” 3 for “general/unknown,” and 5 for “strongly understood/supportive/willing”). We further collected data on the nursing staff's knowledge of the advantages and disadvantages of the multi-dose packaging contrast compared with those of the single-dose packaging contrast.

#### 2.1.3. Evaluation of Operating Time, Number of Operating Times, and Hospital Imaging Capabilities

The operating time and amount of time needed to change the contrast bottle were mainly recorded using a stopwatch. Evaluations were conducted across six steps of the operating procedure: (1) changing injectors (when using dual-syringe PIs), (2) changing catheters, (3) changing the bottle of normal saline, (4) changing the bottle of contrast, (5) exhausting the air out of the bottle and the catheter, and (6) connecting a catheter to the indwelling needle of the patient. To ensure accurate recording, each time point was defined after the pilot survey, and the time period of each process was obtained by subtracting the previous time point from the next time point.  Table 1 presents definitions for each time point. Moreover, the evaluation of hospital imaging capabilities, such as counting the number of CT machines and the annual number of CTs and enhanced scans, was conducted using a questionnaire.

### 2.2. Statistical Analysis

The sample size was calculated using the PASS software, with a power of 80%, significance of 0.05, and level of confidence of 95%. Real-world data, such as the catheter's operating time, were recorded and sorted by the investigators using Excel Spreadsheet 2018. Qualitative data (nursing staff's knowledge and attitude toward different contrast agent specification) were summarised with descriptive statistics using frequency and percentages; quantitative data (nursing staff workflow under different packages of contrast) were summarised using mean and standard deviation. The statistical significance of any difference in the frequency distributions was tested using the two-sample*t*-test and the *χ*^2^ test. Correlation and partial correlation analyses were further performed to assess potential influence of the nursing staff's operating time, knowledge, and attitude toward different dose-response contrast methods. A value of *P* < 0.05 was considered statistically significant. All data were analysed using Stata 15.0 software.

## 3. Results

Between October and November 2018, we observed contrast agent operations among 1,167 patients, specifically 227 patients with 100 ml and dual-syringe PI, 300 patients with 200 ml and dual-syringe PI, 560 patients with 100 ml and syringeless PI, and 80 patients with 200 ml and syringeless PI. A total of 1,032 patients were enrolled, excluding 135 patients whose basic information was not filled out. The patients were gender-balanced, with 53.49% males. The patients' ages ranged from two to 94 years, with the average age being 55 years. Overall, patients weighed 61.4 kg and had an average height of 163.71 cm. Furthermore, we enrolled 42 nurses in eight tertiary hospitals. The nurses' ages ranged from 25 to 64 years, with a mean of 40 years.

### 3.1. Workflow of Nurses and Efficiency of Hospital Scan

#### 3.1.1. Difference of Nursing Staff Workflow under Different Packages of Contrast

To explore the influence of different contrast agent specifications (100 and 200 ml) on the operating time of nurses, this study investigates the differences in nurse operating times under different contrast agent specifications after controlling for the type of PI. Under different packaging contrasts, analyses of each operating procedure indicated that the replacement of contrast agents and the overall nursing staff operating time were the main determinants of workflow efficiency.

Our study results show that contrast agent replacement procedures are less cumbersome with multi-dose packaging contrast compared with single-dose packaging contrast. When a single-dose packaging contrast was used with the dual-syringe PI, the nursing staff conducted 227 contrast-agent bottle replacements in 7.0 days, with an average of 32.43 bottle replacements in a day and 41.97 ± 24.72 s per capita daily time. However, when multi-dose packaging contrast was used, the nursing staff conducted only 135 bottle replacement operations within 7.5 days, with an average of 18.00 bottle changes per day and 19.71 ± 14.79 s per capita daily time, which is significantly shorter than the replacement time reported for using a single-dose packaging contrast (*t* = 12.88, *P* < 0.001). Additionally, contrary to single-dose packaging contrast (which requires replacing a new agent bottle each time, i.e., frequency of change = 1 time/person), the frequency of changing contrast agent was significantly reduced using multi-dose packaging contrast, i.e., 0.45 times/person (*χ*^2^ = 183.44, *P* < 0.001) when delivered by dual-syringe PI and 0.30 times/person (*χ*^2^ = 429.59, *P* < 0.001) when delivered by syringeless PI.

Similarly, overall operating times were more favorable with multi-dose packaging contrasts. When a single-dose packaging contrast was used with a dual-syringe PI, the nursing staff's average overall operating time was 84.64 ± 11.67 s; when the multi-dose packaging contrast was used with a dual-syringe PI, the nursing staff's average overall operating time was 68.47 ± 8.11 s, significantly shorter than the time required for a single-dose packaging contrast (*t* = 18.79, *P* < 0.01). Similar conclusions were made regarding contrast delivery using a syringeless PI. The average operating time of the nurses when a single-dose packaging contrast was used with a syringeless PI was 64.81 ± 19.72 s; the average overall operating time of the nurses when a multi-dose packaging contrast was used together with a syringeless PI was 45.18 ± 0.06 s, significantly shorter than the time required for a single-dose packaging contrast (*t* = 8.90, *P* < 0.01) (Table 2).

Given that different types of PI could influence the overall operating time of nurses, the nurses' operating time for different packaging contrast agents was standardized according to the market ratio of different PIs. According to interviews with key informants, the market ratio of dual-syringe and syringeless power injectors was close to 4 : 1. Therefore, the operating time per capita was inferred to be 80.67 s with a single-dose packaging contrast (100 ml) and 63.81 s with a multi-dose packaging contrast (200 ml), indicating a difference of 16.86 s.

Additionally, based on the 200 ml product contrast agent scenario, our study simulates a 500 ml product contrast agent scenario. Given that the operating time saved was mainly from the replacement of the contrast agent bottle, compared with the 200 ml specification, the 500 ml contrast agent bottle was replaced once for every three patients, and the operating time was reduced to once per cycle. In other words, the decanting time of the 500 ml bottle was half of the time required for a 200 ml bottle. Therefore, the simulation results suggest that, when a multi-dose packaging contrast (500 ml) is used with a syringeless PI, the daily operating time per capita would be 43.83 s. When used with a dual-syringe PI, the daily per capita operating time would be 51.89 s. After standardizing to the market share of different PIs, the daily per capita operating time of the multi-dose packaging contrast (500 ml) would be 50.22 s (Table 3).

#### 3.1.2. Effect of Different Packaging Contrast Agents on Scanning Efficiency

From the hospital's perspective, considering that the time saved by the nursing staff could be used to scan more patients, this study calculates the time saved by using contrast agents of different specifications and the average total scanning time of patients. The daily per capita operating times of the patients using the 200 and 500 ml specifications were 63.81 s and 50.22 s, respectively, and the time savings were 16.86 s and 30.45 s, respectively. Scanning efficiency was also assessed based on the average total scanning time of patients and the annual number of enhanced scans in the hospital. When the annual number of enhanced scans in the hospital was 49,807 (the mean value of this study), the annual increase in enhanced scanning with 200 and 500 ml contrast agents was 2,143 and 4,010 cases, respectively. Daily scans further increased by 7 and 13, respectively (Table 4).

### 3.2. Partial Correlation Analysis of Nursing Staff Operating Time and Related Factors

To further explore the influence of contrast agent specification, the type of PIs, and the daily scanning times on the replacement and overall operating time of contrast agents, this study performs partial correlation analysis under the premise of controlling all other factors; the results are convincing, as shown in Table 3. Partial correlation analysis shows that after controlling the type of PI and the number of daily scans, the contrast agent specification (0 = 100 ml, 1 = 200 ml) and the replacement time of the contrast agent were significantly correlated. In other words, the larger the contrast agent specification, the shorter the corresponding replacement time (Table 5).

### 3.3. Knowledge, Attitude, Willingness to Use, and Experience of Nurses

Among the 42 nurses enrolled, a higher proportion (83.33%) of the nursing staff thought that they were very or quite familiar with the multi-dose packaging contrast, 59.53% supported the use of the multi-dose packaging contrast, 57.14% expressed willingness to use the multi-dose packaging contrast to carry out relevant work, and 11.9% expressed strong willingness to use the multi-dose packaging contrast.

The nursing staff's knowledge of the advantages and disadvantages of the multi-dose packaging contrast compared with that of those of the single-dose packaging contrast was also examined. Among the 29 nurses (69.05%) who responded about the comparative advantage of the multi-dose packaging contrast, the first three answers were “saves operating time” (*n* = 9, 31.03%), “reduces operating times” (*n* = 9, 31.03%), and “allows more convenient operations” (*n* = 6, 20.69%). Among the 28 nurses (66.67%) who responded about the relevant disadvantages, the top two answers were “inconvenient for patients to prescribe drugs and charge fees” (*n* = 5, 17.86%) and “if the contrast agent is contaminated, many patients will be affected” (*n* = 3, 10.71%).

## 4. Discussion

Medical diagnostic imaging, especially enhanced CT scanning, is an important health technology for disease diagnosis. Many physicians and patients prefer enhanced CT scanning to better detect diseases and improve imaging quality, resulting in the extensive use of contrast agents. Over the last few decades, IV CECT has only been available in single-dose packages. When using single-dose packages, nursing staff are required to conduct an entire operating process which includes changing the contrast agent bottle for each enhanced CT scan. Not only were the workflow and workload of the nursing staff complicated and heavy but also hospital efficiency was often affected, resulting in long waiting times for enhanced CT scanning. This is the first study to evaluate the effect of different contrast agent specifications on the nursing staff's workflow, operating time, knowledge, and hospital efficiency based on the different types of PIs and therefore can serve as a reference for clinical and hospital-based decisions.

We divided the contrast agent injection workflow of the nursing staff into six main steps: changing injectors, changing catheters, changing the bottle of normal saline, changing the bottle of contrast, exhausting the air out of the bottle and catheter, and connecting the catheter to the indwelling needle on patients. According to the quantitative results, the number of contrast bottle changes and the time consumed can be reduced, and the steps for injecting the contrast agent can be simplified. Assuming that nursing staff adopt the multi-dose packaging contrast and the work pace remains unchanged, hospital income may increase, thereby improving the nursing staff's performance evaluation index and work performance. If the number of yearly enhanced scans in the hospital remains stable, nursing staff can also relatively use the time saved to appropriately reduce their work pace, fatigue, and tension caused by their high-intensity workloads.

This study demonstrates that a 200 or 500 ml contrast agent could reduce CT wait times and overall hospital workflow burden. Typically, registering for a CT examination on the same day is usually impossible owing to the large number of outpatients and inpatients undergoing enhanced scanning; in fact, patients must book an appointment one to two days in advance or even earlier. Following the use of a multi-dose packaging contrast agent, however, the nursing staff's operating time can be reduced, thereby reducing the total time for enhanced scanning. On the one hand, the CT wait times for patients can be reduced. On the other hand, hospitals can use the time saved to perform enhanced CT scans on more patients, thereby improving hospital workflow under the same work intensity and further increasing hospital economic benefits.

The type of PI could also affect the nursing staff's operating time. Unlike the dual-syringe PI, the syringeless PI can automatically extract the contrast agent from the bottle without manual suction [11, 15], thereby significantly reducing the operating time with a 200 ml contrast agent. However, considering the high cost of syringeless PIs, this study only suggests popularizing the multi-dose packaging contrast and the syringeless PI in appropriate medical institutions, namely, hospitals with more rather than fewer outpatients.

Responses from the nursing staff at the eight tertiary hospitals analysed in this study showed that more than half of the nursing staff exhibit positive sentiments toward the degree of understanding, attitude, and intention of use for multi-dose packaging contrasts. The correlation analysis results showed a significant correlation between degree of understanding, attitude tendency, and intention of use, further confirming the consistency of the nursing staff's positive sentiment toward multi-dose packaging contrasts. Regarding understanding the advantages and disadvantages of the multi-dose packaging contrast (compared with the single-dose packaging contrast), 19 of the 28 respondents mentioned the word “waste,” with part of the answer being based on the “one person one bottle case,” i.e., using one bottle of the multi-dose packaging contrast for only one patient results in wastage. Although 83.33% of the nursing staff had knowledge of the contrast agent according to the self-rating scale, the realistic clinical setting may be different. By popularizing the use of multi-dose packaging contrasts, we can help reduce gaps in the nursing staff's knowledge and relatively improve their attitude and acceptance degree.

However, this study has some limitations. First, currently, formal standardized operating procedures on contrast agents do not exist, resulting in different sequences of operating steps for nursing staff. Although evaluating operating procedures became more difficult, we divided the entire operating process into specific steps and timed each step. Second, compared with the scanning volume of the 100 ml contrast agent specification using a syringeless PI, the scanning volume of the 200 ml contrast agent was 80 patients. Accordingly, the sample size of 100 ml contrast agent specification scans may need further research to verify the results of this study. Lastly, considering the relatively small total sample size (i.e., only eight sampled hospitals), the extrapolation of this study may have certain limitations; if the study results are to be extrapolated, there may be a need to evaluate a larger sample in future research.

## 5. Conclusion

Through difference and correlation analysis on real-world data, this study suggests that, considering safety, the use of a multi-dose bulk IV contrast agent is more time-saving and efficient for Chinese nurses and medical institutions compared with that of a single-dose package. These results may provide important references for clinical and hospital-based decisions involving contrast media utilization in China.

## Figures and Tables

**Table 1:** Definition of each time point.

Process	Time point	
	Previous time point	Next time point
Changing injectors	The moment the nurse's hand touches the old syringe of a dual-syringe PI and prepares to change the injector	The moment the nurse's hand releases the new syringe of the dual-syringe PI and completes changing the injector
Changing catheter	(1) Changing the bottle of normal saline or contrast: the first moment is when the nurse's hand touches the medical calm or the joint of the catheter and the injector, and the second moment is when the old bottle is placed on the table	(1) Changing the bottle of normal saline or contrast: the first moment is when the nurse's hand touches the new bottle, and the second moment is when the nurse's hand touches the button on the PI and prepares to exhaust the air
	(2) Not changing the bottle: the moment the nurse's hand touches the joint of the catheter and the injector	(2) Not changing the bottle: the moment the nurse's hand touches the button on the PI and prepares to exhaust the air.
Changing the bottle of normal saline/contrast	The moment the nurse's hand touches the new bottle	The moment the old bottle is placed on the table
Exhausting the air out of the bottle and catheter (only the waiting time of the nurses for the end of exhaust is recorded; the exhaust time for synchronous catheter change and other operations will not be recorded.)	(1) Dual-syringe PI: the moment the nurse's hand touches the button on the panel of the PI or touches the knob on the bottom of the PI	(1) Dual-syringe PI: the moment the nurse's hand touches the indwelling needle of the patient or the catheter is placed on the shelf
	(2) Syringeless PI: the moment the nurse's hand touches the button on the panel of the PI	(2) Syringeless PI: the moment the machine makes no sound
Connecting the catheter to the indwelling needle of the patients	The moment the nurse's hand touches the indwelling needle of the patient	The moment the nurse's hand releases the indwelling needle of the patient

**Table 2:** Workflow of nursing staff with different packaging contrasts.

Type of power injector	Contrast agent specification (ml)	Operation of exchanging contrast agents			Overall operating time
		Time (s, average (standard deviation))	Number of times (times)	Frequency (times/person)	
Dual-syringe power injector	100	41.97^*∗∗∗*^ (24.72)	227	1.00^*∗∗∗*^	84.65^*∗∗*^ (11.67)
	200	19.71^*∗∗∗*^ (14.79)	300	0.45^*∗∗∗*^	68.47^*∗∗*^ (8.11)
Syringeless power injector	100	19.39 (18.45)	560	1.00^*∗∗∗*^	64.81^*∗∗*^ (19.72)
	200	23.43 (2.60)	80	0.30^*∗∗∗*^	45.18^*∗∗*^ (0.06)

^^*∗*^^ *P* < 0.05, ^^*∗∗*^^*P* < 0.01, and ^^*∗∗∗*^^*P* < 0.001.

**Table 3:** The daily per capita operating time of patients using different specifications of contrast agents.

Specification (ml)	Type of power injectors	Cases	Daily per capita operating time (s)	Market share of different PIs (%)	Daily per capita operating time(*s*)^*∗*^	*t*	*P*
100	Syringeless	560	64.81	20	80.67	6.28	<0.001
	Dual-syringe	227	84.64	80			
200	Syringeless	80	45.18	20	63.81		
	Dual-syringe	302	68.47	80			
500	Syringeless	—	43.56	20	50.22	—	—
	Dual-syringe	—	51.89	80		—	—

^^*∗*^^: adjusted by market share of PIs.

**Table 4:** Effect of different specifications of contrast agents on hospital efficiency.

Annual number of enhanced scans in the hospital	Specification (ml)	Daily per capita operating time (s)	Time saving per capita (s)	Average total scan time of patients (s)	Annual increase in enhanced scanning (# cases)	Daily increase in scanning
Average (49,807)	100	80.67	—	408.63	—	—
	200	63.81	16.86	391.77	**2143**	**7**
	500	50.22	30.45	378.18	**4010**	**13**
Minimum (15,000)	100	80.67	—	408.63	—	—
	200	63.81	16.86	391.77	**646**	**2**
	500	50.22	30.45	378.18	**1207**	**4**
Maximum (70,470)	100	80.67	—	408.63	—	—
	200	63.81	16.86	391.77	**3033**	**10**
	500	50.22	30.45	378.18	**5674**	**19**

**Table 5:** Partial correlation analysis for the replacement time of contrast agents and the overall operating time.

Relative factors (ml)	Partial correlation coefficient	
	Replacement time of contrast agents	Overall operating time
Contrast agent specification (100 = 0, 200 = 1)	−0.44^*∗∗*^	−0.43^*∗∗*^
Type of power injector (dual-syringe = 0, syringeless = 1)	0.84^*∗∗*^	0.52^*∗∗*^
Daily scans (no.)	0.17^*∗∗*^	−0.03

^^*∗*^^ *P* < 0.05, ^^*∗∗*^^*P* < 0.01, and ^^*∗∗∗*^^*P* < 0.001.

## Data Availability

The data used to support the findings of this study are available from the corresponding author upon request.
